# Symmetrical and Peripheral Gangrene Complicating a Third-Degree Atrioventricular Block: A New Presentation of a Known Disease

**DOI:** 10.7759/cureus.10477

**Published:** 2020-09-16

**Authors:** Hanane Aissaoui, Karima Benbouchta, Noha Elouafi, Brahim Housni, Nabila Ismaili

**Affiliations:** 1 Cardiology, Mohammed I University/Mohammed VI University Hospital/Epidemiological Laboratory of Clinical Research and Public Health, Oujda, MAR; 2 Intensive Care and Anesthesiology, Mohammed I University/Mohammed VI University Hospital/Epidemiological Laboratory of Clinical Research and Public Health, Oujda, MAR

**Keywords:** case report, symmetrical and peripheral gangrene, low cardiac flow, third degree atrioventricular block

## Abstract

Symmetrical peripheral gangrene (SPG) is a rare, serious entity characterized by ischemic changes of the distal extremities with no vessel occlusion, leading to fatal complications. It is related to numerous causes, and the treatment is not yet consensual. We present the first case of SPG related to low cardiac output secondary to a third-degree atrioventricular block. Physicians should be aware of this entity, as early recognition and adequate management can help in reducing morbidity and mortality and prevent fatal complications.

## Introduction

Symmetrical and peripheral gangrene (SPG) is a rare entity. It was first illustrated by Hutchinson in 1891 [1]. It is defined by a sudden onset of symmetrical ischemia of two or more extremities, leading to gangrene in the absence of large vessel obstruction or vasculitis, increasing the risk of limb amputation and affecting the quality of life [2-3]. The etiology is multifactorial. Several infective and non-infective etiologies were related to SPG [4]. It is associated with sepsis, low cardiac output states, hyperviscosity syndromes, chronic myeloproliferative, and vasospastic disorders [5]. The inappropriate use of vasoactive drugs has been also described. We report here the first case of severe four limbs SPG secondary to an atrioventricular block (AVB).

## Case presentation

**Figure 1:**
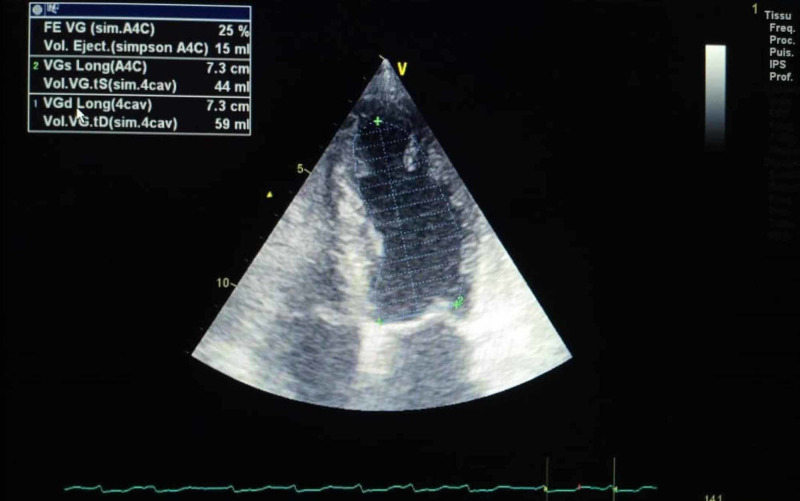
Transthoracic echocardiography showing severe LV systolic dysfunction EF 25% LV: left ventricle; EF: ejection fraction

**Figure 2:**
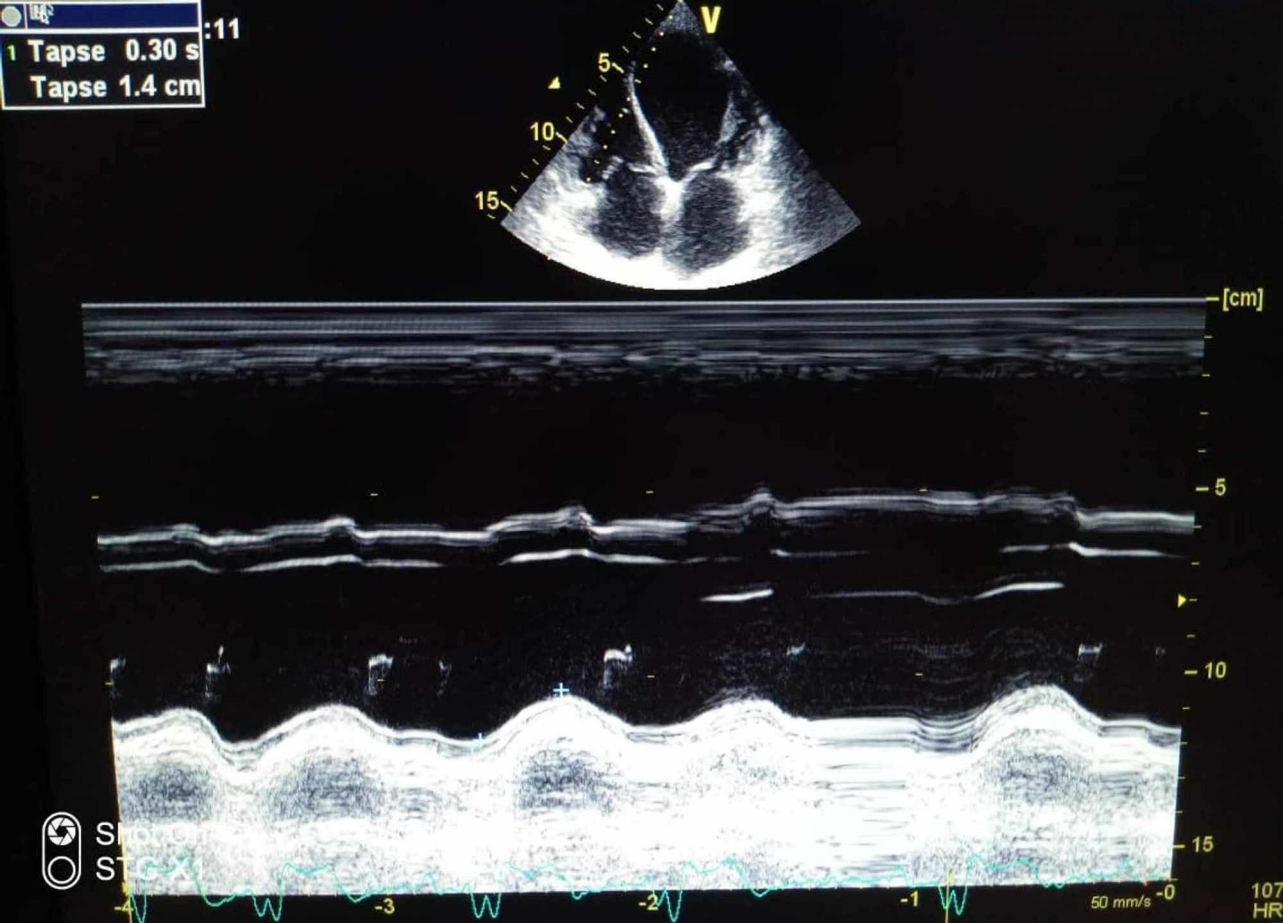
Transthoracic echocardiography showing RV systolic dysfunction RV: right ventricle

**Figure 3:**
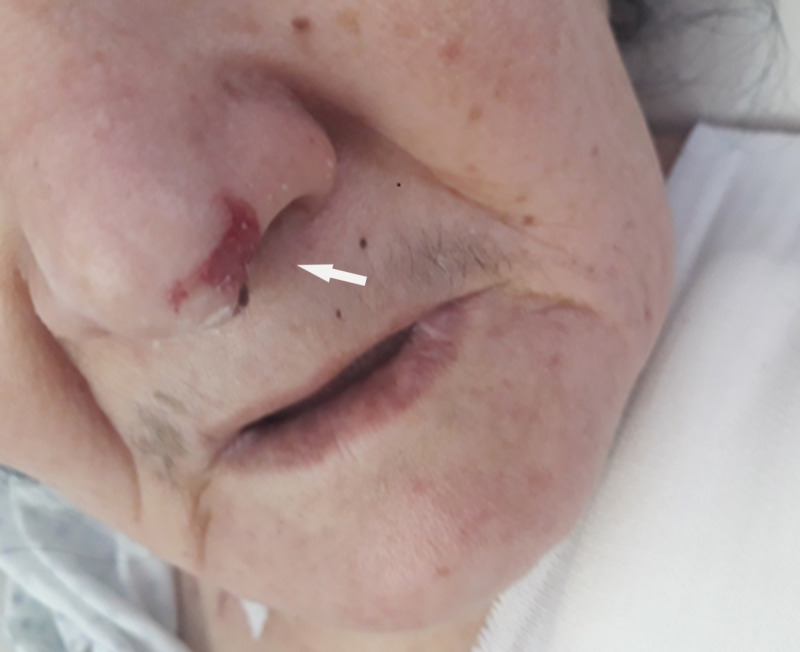
Discoloration of the tip of the nose

**Figure 4:**
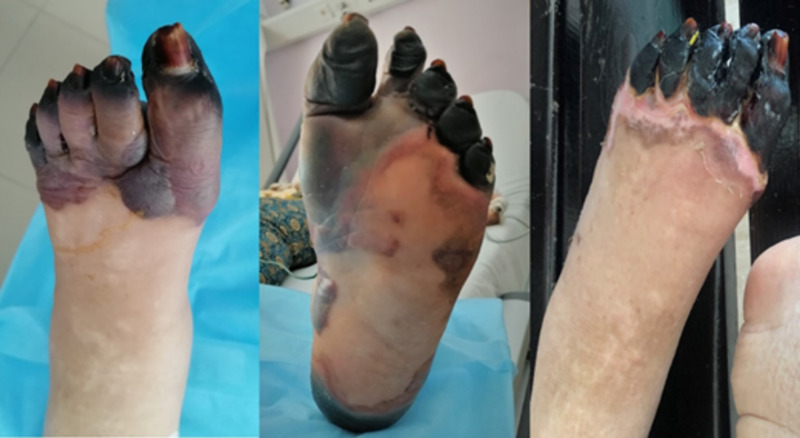
Dorsal and palmar aspects of the left foot

We report the case of a 73-year-old female, with a history of diabetes, hypertension, dyslipidemia, and a complete heart block three months before. The patient had refused pacemaker insertion. She was admitted for repetitive syncope. At presentation, she was unconscious; her pulse was 20 beats per min; her blood pressure was 70/30 mmHg, and her respiratory rate was 17 per min; maintaining oxygen saturation at 96% on room air; her temperature was 36.7°C. Heart auscultation revealed bradycardia. Examination revealed cyanosis of the upper and lower extremities. After her admission, the patient suddenly presented asystole requiring cardiopulmonary resuscitation (CPR). Return of spontaneous circulation (ROSC) was observed after five minutes of CPR and mechanical ventilatory support was instituted. The electrocardiogram showed a third-degree atrioventricular block with a ventricular rate at 20 bpm. Transthoracic echocardiography (TEE) revealed a low ejection fraction (EF) of 25%, with segmental kinetic disorders, and mild right ventricular systolic dysfunction; no left ventricle (LV) thrombus was noted. The patient was started on vasopressor infusions (dobutamine: 10 mcg/kg/min), and norepinephrine 0.4 mcg/kg/minute) to support systolic blood pressure. She was admitted then to the catheterization lab for temporary transvenous pacing. The angiogram showed moderate ostial stenosis of the posterior interventricular artery, and severe stenosis of the middle segment of the left anterior descending artery, needing a myocardial ischemia test. Dual-chamber permanent pacemaker implantation was performed successfully. Laboratory investigations revealed impaired renal and liver functions, an elevated white blood cell count (14.000/µL), and a normal platelet count (192.000 /µL). Prothrombin time was normal. The lactate level was elevated (8 mmol/L). Due to hemodynamic improvement, all vasopressors agents were gradually weaned off and eventually discontinued after two days. Mechanical ventilation support was discontinued. Yet, we noted worsening of the skin discoloration on the patient's upper and lower extremities and on the tip of her nose (Figures 1-4), with prolonged capillary refill time.

**Figure 5:**
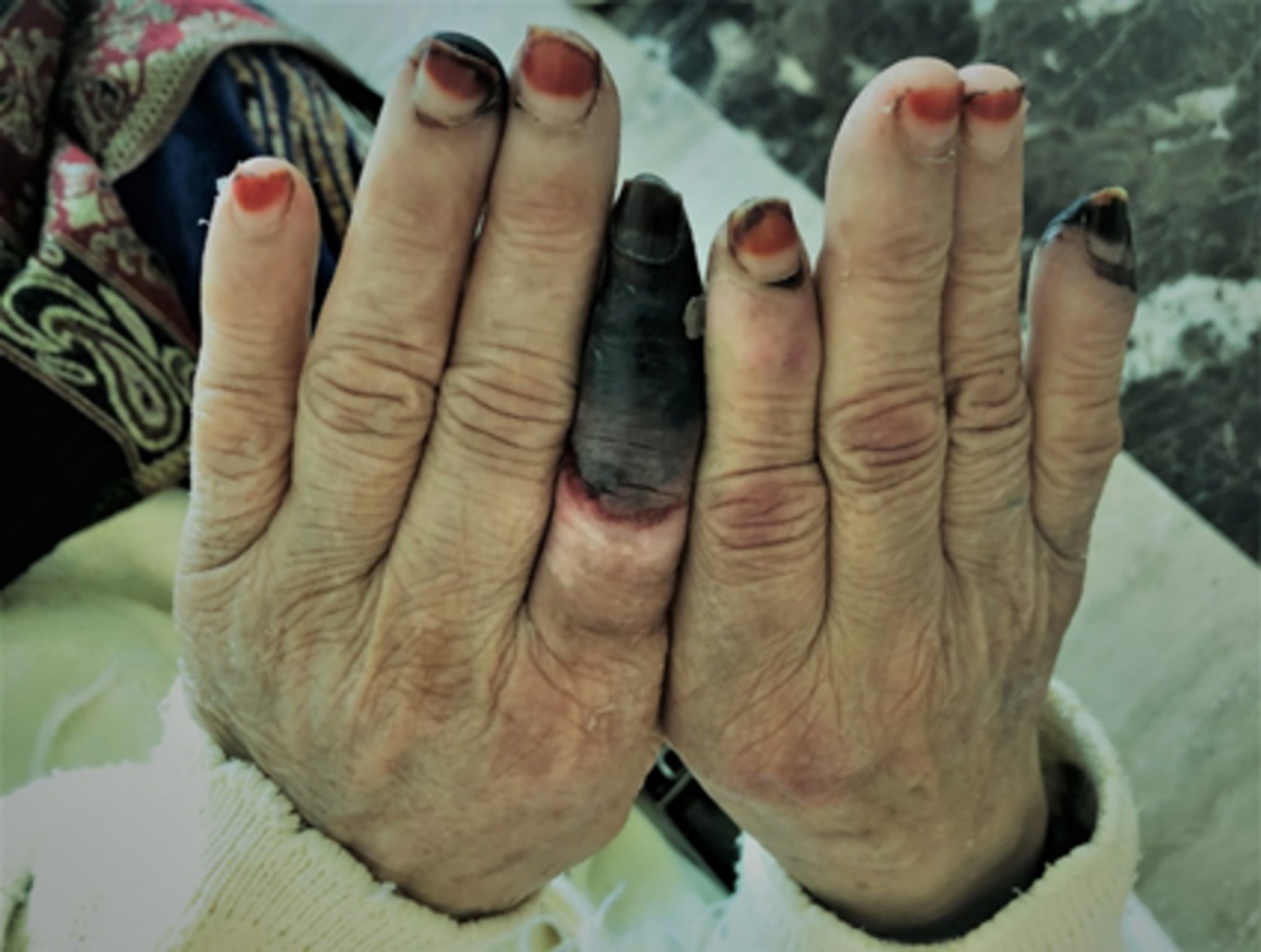
The dorsal aspect of both hands

**Figure 6:**
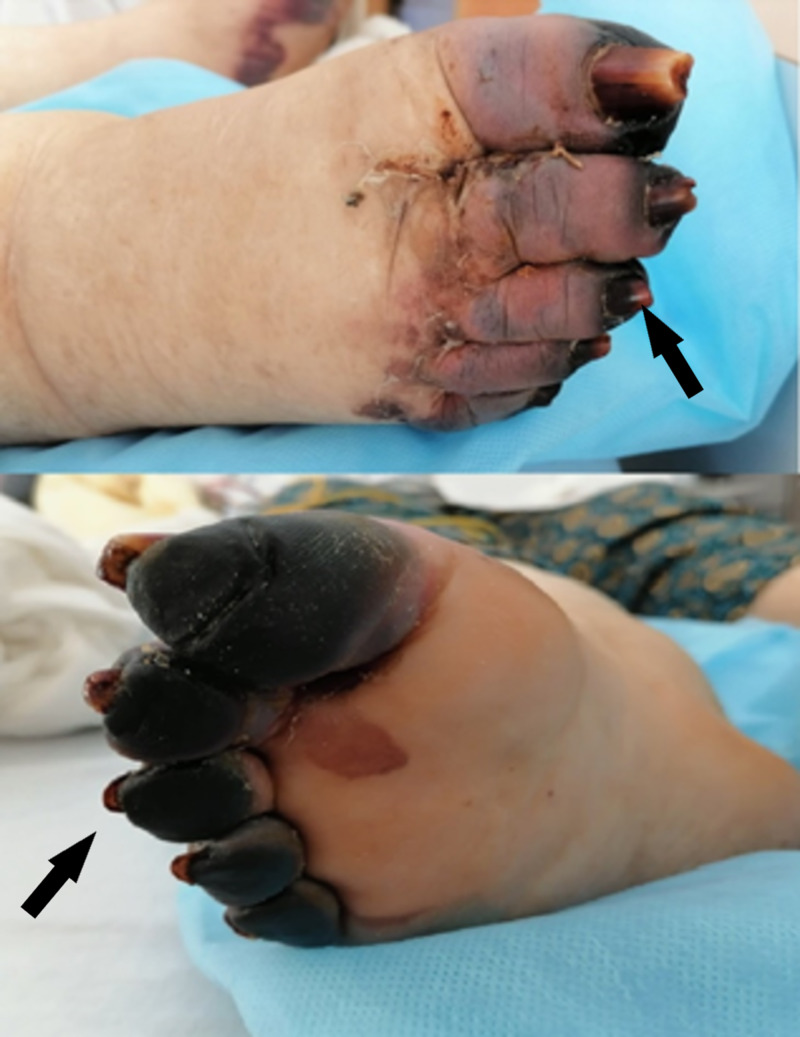
The dorsal and palmar aspects of the right foot

**Figure 7:**
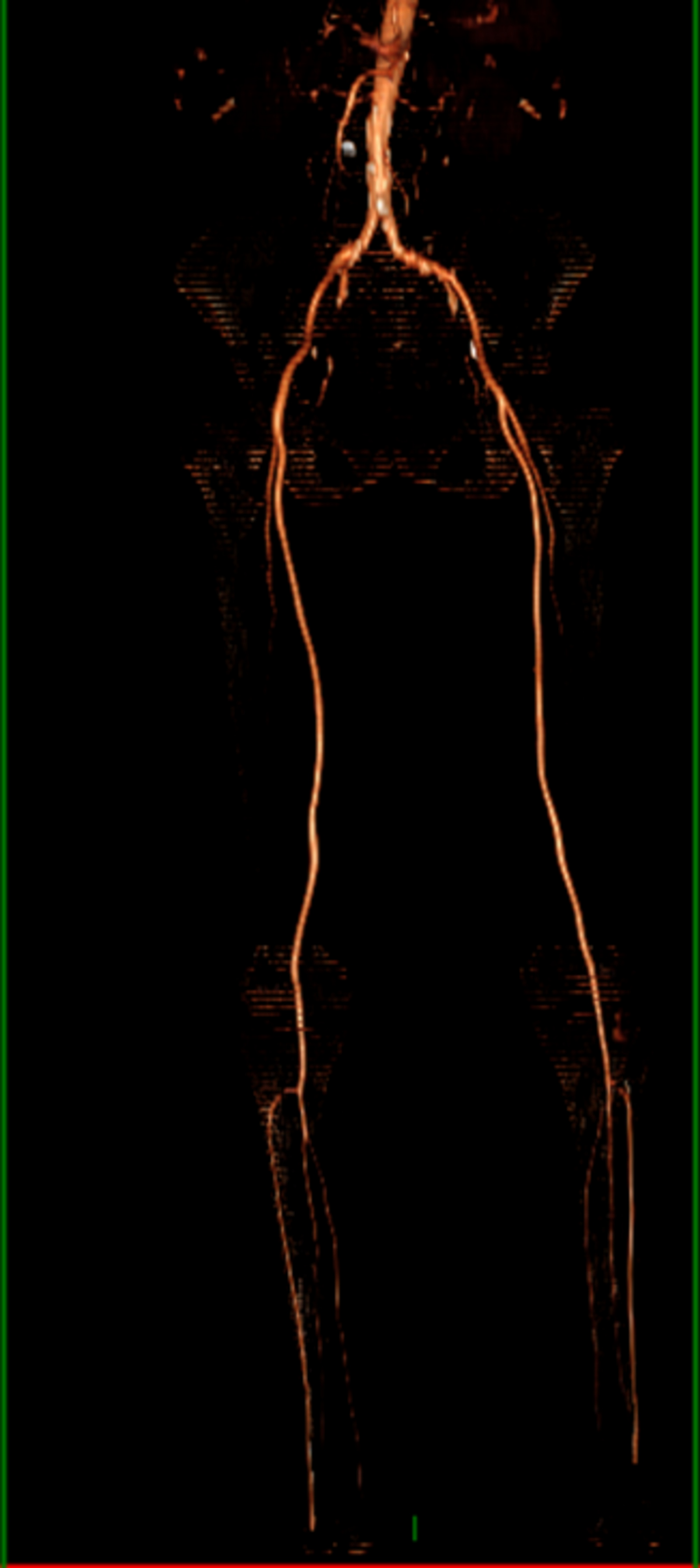
CT angiography of the aorta and its branches with no significant stenosis CT: computed tomography

All peripheral pulses were normally palpable. Capillaroscopy showed no abnormalities; no other relevant findings on physical examination were found. Three days later, the patient developed gangrenous changes to her left index, her right little finger, and the toes of both feet (Figures 5-6). On Day 9 since admission, the patient’s feet contained fluid-filled bullae with gangrenous changes. Doppler ultrasonography revealed normal blood flow of the lower extremity arteries. We completed computed tomography (CT) angiography of the aorta and its branches (after normalization of renal function), which did not show any significant stenosis (Figure 7).

Skin biopsy showed non-specific inflammatory changes and ruled out vasculitis as a potential cause of this presentation. Extensive medical investigations were conducted, and differential diagnoses were ruled out. Based on these features, we diagnosed symmetrical peripheral gangrene probably due to low cardiac flow secondary to a third-degree atrioventricular block, worsen by the use of vasoconstrictor drugs. Our patient was placed on a converting enzyme inhibitor, calcium channel blocker, a low dose of heparin, and the local application of heparin ointment. On her 15th hospital day, despite antibiotic and anticoagulant therapy, the acral necrosis and dry gangrene with mummification remained. The affected extremities were protected from further trauma, cold, and secondary infection. The patient was discharged from the hospital after having been given medical, vascular, and plastic surgery outpatient department appointments.

## Discussion

SPG is an uncommon clinical revelation of a sudden onset of symmetrical ischemia in two or more extremities in the absence of proximal arterial obstruction and vasculitis [4-5]. Fingers and toes are most frequently affected [5]. The exact mechanism for SPG is unknown [6], and it has been associated with multiple infective and non-infective aetiologies and can affect any age or either sex [6]. This condition may result from acute bacteremia or vasopressor drug use, which results in the occlusion of microcirculation of the affected part. Many cardiac conditions were associated with SPG such as myocardial infarction [7-10], ventricular pseudoaneurysm [11], pulmonary embolism [12], paroxysmal ventricular tachycardia [13], post-cardiac surgery [14-15], severe heart failure [3], and postpartum cardiomyopathy (Table 1) [16]. It may be also encountered in some chronic conditions such as essential thrombocythemia, polycythemia rubra vera, Raynaud's syndrome, diabetes, small vessel obstruction, and disseminated intravascular coagulation (DIC) [7]. Many reports suggest DIC as the common pathway of SPG pathogenesis [6].

**Table 1:** Different cardiac conditions related to SPG SPG: symmetrical peripheral gangrene

Age	Sex	Condition	mechanism	The Day of ischemia	Clinical presentation	Outcome	Authors
53	M	Acute myocardial infarction	Low cardiac output (Cardiogenic shock)	Day 6	Both feet	Death	Wg.Swan, et al [7]
64	M	Acute myocardial infarction	Low cardiac output (Cardiogenic shock)	Day 4	Both feet	Amputation Death	Robert T, et al [8]
73	M	Myocardial infarction	Low cardiac output (Cardiogenic shock)	Day 3	The tips of the fingers and toes. The tip of the nose	Death	Harold Cohen, et al [9]
58	F	Myocardial infarction	Low cardiac output (Cardiogenic shock)	Day 4	Tip of the nose, fingers, toes	Death	S. J. Caserta, et al [10]
54	F	Ventricular pseudoaneurysm	Low cardiac output, and acute left ventricular failure associated with DIC	Day 4	Feet and fingers	Death	Sudip Kumar Ghosh, et al [11]
51	M	Pulmonary embolus	Circulatory collapse, poor oxygenation	Day 10	The ears, nose, lips Hands, and feet.	Amputation Survival	Milton.R, et al [12]
47	F	Persistent ventricular tachycardia	Low cardiac output	Day 2	The hands, feet, nose, and ears	Death	D. Gordon Abrahams, et al [13]
63	F	Symmetrical peripheral gangrene associated with cardiac surgery	Borderline cardiac output (3.8 l/min, cardiac index (2.1 l/min/m2).	Day 2	Fingers and toes	Amputation and survival	Rajinder Singh, et al [14]
43	F	Symmetrical gangrene of following the insertion of a Starr-Edwards mitral valve prosthesis into a patient with a giant left atrium	Low cardiac output	Day 4	The fingers and legs	Amputation and survival	James L. Guest, et al [15]
64	M	Low output cardiac failure (FE 10%; cardiac index of 1.28 l/min/sqm)	Dobutamine infusion and diuretics	Several days before admission	Toes of both feet	Amputation and survival	Sijan Basnet, et al [3]
37	F	Peripartum cardiomyopathy (FE 40%)	Low cardiac output	7 days post-partum	Toes	Amputation Survival	Ajay Jaryal, et al [16]

Our patient was not septic on presentation and did not have a clear source of infection that might have led to DIC but had a three-months history of third-degree AVB causing a reduction of the blood flow through digital arteries. A low-flow state can lead to ischemic changes. Aggravating factors include the use of vasoconstrictor drugs [2-3], diabetes mellitus, and renal failure [2].

The diagnosis of SPG is suspected within patients with dusky discoloration of extremities and high lactate levels. Doppler ultrasonography of the extremities may reveal normal peripheral pulses. CT-angiography of the aorta and its branches may show no stenosis images [14]. Occlusion of the small vessels can be found on a skin biopsy.

The treatment of GPS is not yet consensual. Early recognition of SPG, hemodynamic stabilization using intravenous fluids, and the management of DIC and the underlying etiology are essential to determine the outcome [17].

The use of vasodilators such as intravenous nitroprusside and a local or intravenous infusion of alpha-blockers (phentolamine, chlorpromazine) may be helpful. Phentolamine is an α-adrenergic blocker, which is known for its central nervous system effect and may increase the cutaneous blood flow [6]. Nitroglycerin ointment is a topical vasodilator that has been proven to improve the skin condition. Intravenous prostacyclin (epoprostenol) has also been reported as beneficial. The use of IV antibiotics and low-dose heparin treatment is beneficial to treat sepsis syndrome and DIC.

The treatment by sulodexide has been proven effective. It a purified glycosaminoglycan, characterized by its antithrombotic, profibrinolytic, and vasodilator properties, to diminish platelet aggregation and improve circulation, viscosity, and arterial blood flow to relieve pain and trophic changes [17].

Local debridement, amputation of the gangrenous areas, and skin grafting may be necessary. Early physiotherapy may facilitate the regaining of function and rehabilitation [18].

Our patient was placed on an angiotensin-converting enzyme inhibitor, calcium channel blocker, a parenteral low dose of heparin, and the local application of heparin ointment. Luckily for our patient, the use of a vasopressor drug was discontinued early, and she underwent a pacemaker placement to improve her blood flow.

Unfortunately, SPG carries a poor prognosis. The mortality rate is estimated at 10% to 30% of cases [19]. Most deaths occur within five to 21 days after the onset of gangrene [4]. Amputation should be considered only after the development of a demarcation separating the healthy zone from the necrotic zone [18-19].

## Conclusions

SPG is associated with significant mortality and morbidity, with a frighteningly high rate of amputation amongst survivors. Early recognition and adequate management are vital to avoid this condition. In all cases, the rapid correction of tissue hypoperfusion and rapid etiological treatment allows an improvement in the prognosis.
